# Designing App Interfaces to Elicit Specific Emotional Responses and Improve Attention and Short-Term Memory in Patients With Insomnia Undergoing Brief Cognitive Behavioral Therapy: Within-Subject Eye-Tracking Experimental Pilot Study

**DOI:** 10.2196/79883

**Published:** 2026-02-19

**Authors:** Kuan-Chu Su, Hsiao-Yean Chiu, Ko-Chiu Wu, Chia-Chi Chang

**Affiliations:** 1 College of Design, National Taipei University of Technology, Taipei, Taiwan; 2 School of Nursing, College of Nursing, Taipei Medical University, Taipei, Taiwan; 3 Department of Nursing, Taipei Medical University Hospital, Taipei, Taiwan; 4 Research Center of Sleep Medicine, College of Medicine, Taipei Medical University, Taipei, Taiwan; 5 Research Center of Sleep Medicine, Taipei Medical University Hospital, Taipei, Taiwan; 6 Department of Interaction Design, National Taipei University of Technology, Rm.701-4, Design Building, No.1, Sec.3, Chung-Hsiao E. Rd, Taipei, 10608, Taiwan, 886 912 595408, 886 2 87732913

**Keywords:** cognitive behavioral therapy, emotional design, memory, eye tracking, attention, interface design, insomnia

## Abstract

**Background:**

Patients with insomnia have difficulty in both falling asleep and maintaining sleep. Individuals with long-term sleep deprivation are prone to poor concentration and impaired memory; however, these problems can be alleviated following brief behavioral treatment for insomnia (BBT-I). This study involved the design of an app called “Sleep Well” that enables individuals with insomnia to easily record their sleep behavior. The app guides users to recall and record sleep-related information, acquire sleep hygiene knowledge, and communicate with therapists online.

**Objective:**

This study examined how specific sleep diary interface design features in a brief cognitive behavioral therapy for insomnia (BBT-I) app influence users’ attention and short-term memory. Using a combination of objective eye-tracking measures and subjective attention assessments, the study compared 3 interface designs to determine how visual layout, input modality, and interaction style interact with insomnia symptoms to affect attentional performance, memory accuracy, and user preference.

**Methods:**

Three sleep diary interfaces were designed, varying background mode (day vs night), color scheme (blue vs green), box shape (circular, rounded rectangular, or rectangular), and input method (slide-in, tap, or type-in). A total of 33 participants completed standardized diary-entry tasks while eye movements were recorded using an eye tracker to capture gaze trajectories and visual attention patterns during app interaction. User experience, subjective attention, and interface preferences were assessed using structured questionnaires. Data were analyzed using descriptive statistics, nonparametric tests, Pearson correlation analysis, cross-tabulation analysis, and exploratory factor analysis to examine associations among interface design, attentional performance, memory accuracy, and user characteristics.

**Results:**

A total of 33 participants (n=13, 39.4% male and n=20, 60.6% female) aged 20 to 64 years completed this study. Based on the Insomnia Severity Index, 6 of 33 (18.2%) participants had clinical insomnia and 13 of 33 (39.4%) reported insomnia symptoms. Most participants reported staying up late (22/33, 66.7%), and more than half of participants reported drinking tea (17/33, 51.5%). Interface design significantly influenced objective attentional performance, as measured by eye-tracking indicators of task efficiency and visual allocation. Sleep quality and insomnia symptoms were consistently associated with attentional and short-term memory outcomes, with memory accuracy varying across interfaces and showing particular sensitivity to sleep maintenance difficulties. Subjective attentional control was strongly associated with both eye-tracking metrics and memory performance, and interface preferences differed by insomnia status.

**Conclusions:**

Interface design significantly modulates attention and short-term memory performance in users with insomnia. Eye-tracking revealed that insomnia symptoms and sleep quality influence visual attention and task efficiency, whereas subjective attentional control showed stronger and more consistent associations with memory accuracy than physiological eye-movement indicators. These findings suggest that cognitive processing during sleep diary completion relies more on internal attentional states than on observable gaze behavior. Designing low-load, attention-supportive interfaces may therefore improve usability and data accuracy in digital BBT-I interventions.

## Introduction

### Background

Good sleep quality (SlpQ) not only improves learning, memory, retention, and recall, but it also facilitates the creation and innovation of solutions from new information [1]. Sleep further consolidates memories by transferring them from short-term to long-term memory. Sleep deprivation can affect the basal state of attention when performing simple tasks [2]. In addition to affecting memory consolidation, sleep deprivation also reduces attention, alertness, concentration, and problem-solving ability, thereby affecting work and daily life [3-6].

Cognitive behavioral therapy is currently the recommended first-line treatment for insomnia [7]. However, nearly half of patients prefer to receive such treatment online, which is often more convenient and efficient [8]. Cognitive behavioral therapy for insomnia (CBT-I) has been applied to different cohorts of patients with insomnia, such as mobile phone app–based CBT-I for adolescents of different genders [9] and internet-based CBT-I for patients with insomnia who continue to experience symptoms after drug treatment [10]. As mobile technology becomes more integrated into daily life, research on the use of mobile phones to treat insomnia has increased. In addition to strengthening sleep retraining through smartphones [11], CBT-I apps and platforms have also been developed, and the usability of such systems has been thoroughly reviewed [12].

Brief cognitive behavioral therapy for insomnia (BBT-I) retains the behavioral therapy component of CBT-I but shortens the treatment duration from approximately 6 to 8 weeks to just 4 weeks. BBT-I and CBT-I share the use of 2 key behavioral principles (ie, sleep restriction and stimulus control) to enable patients to sleep in a predictable and reliable manner. Sleep restriction limits the time a person spends in bed, whereas stimulus control refers to creating a clear and positive association between bed and sleep.

Owing to the shortened treatment time, BBT-I has been widely adopted for insomnia treatment. The benefits of BBT-I are comparable to those of CBT-I [13]; thus, BBT-I has been suggested as an effective intervention for insomnia management [7]. However, accurate information recording by patients with insomnia and clinicians remains key to effective treatment.

During the COVID-19 pandemic, researchers developed a digital brief therapy for insomnia and explored its adherence and efficacy [14]. In this treatment, patients with insomnia are required to operate a mobile phone interface, recall details of their daily life and sleep from the previous day, and complete a sleep diary. Individuals with insomnia therefore prefer specialized interface designs that allow them to maintain control of their pace of life and sleep patterns [15].

Because diagnostic criteria for categorizing insomnia and cognitive measures vary widely, there is a need to standardize methods for assessing insomnia and cognitive performance in research [5]. Currently, there is limited research on the usability of BBT-I app interfaces for information input and retrieval among patients with insomnia who experience memory and attention impairments.

### Interface Design and Cognitive Considerations in Insomnia

According to the theory of working memory, individuals can remember only 4 to 6 pieces of information within a 20-second timeframe. In mobile application design, the commonly used waterfall layout may lead to reading times that exceed the limitations of human memory. In previous work, we employed a grid layout to highlight important information and demonstrated significant improvements in interface content memory [16]. Moreover, symmetry, order, simplicity, and regularity of interface layout reduce the difficulty of memory formation [17]. These qualities enhance esthetics, facilitate memorization, and reduce cognitive load. When developing an interface, designing elements symmetrically and arranging them in an ordered pattern based on size are recommended. In particular, aligning the edges and starting points of elements as much as possible also improves memorization.

From a physiological perspective, reading in dark mode under low ambient lighting reduces eyestrain. Visual fatigue in nighttime environments is related to color and screen brightness, such that lower screen brightness can reduce visual fatigue. In contrast, under normal lighting conditions, there appear to be no differences between using dark or light mode [18].

The colors green, blue, and black result in the least visual fatigue under low screen brightness conditions, whereas red results in the highest visual fatigue. In a study reviewing the effects of color on visual fatigue, blue was rated as the most favorable color for overall interface design [19]. Blue conveys calmness and stability, white conveys purity and peace, and green is considered a healthy color that can reduce stress and anxiety and elicit positive emotions [20,21].

Interface shape is an important factor in the emotional responses to interface design [22]. Many studies have used corner shape (eg, circular vs angular) to reduce cognitive load in the brain and obtain a better user experience. Patients rate circular elements higher because circles convey tenderness and caring emotions, whereas squares evoke a stronger sense of power, which may create pressure and is less likely to inspire positive emotions [23]. The use of boxes can change the spatial distribution of attention, affect the apparent size of objects, change potential visual perception, and thus affect user attention, maintain device controllability, and reduce cognitive load [24,25].

For the design of a medical app interface, a previous study recommended that the page be operated using click and slide gestures and that instant suggestions be provided during the operation. To enhance readability, clear text in a font size no smaller than 14 points should be used. To distinguish function buttons, a blue-and-white background is recommended, and placing an icon in the upper-left corner of the page can improve visibility. This ensures that there is sufficient distance between function keys for user operations [26].

### Eye Tracking and Cognitive Measures in Interface Evaluation

During information retrieval, eye movements, particularly fixation rate, are predictive for recovering previously remembered details but not for newly generated details. This suggests that during memory recall, the primary function of eye movements is to reproduce already perceived information rather than to generate entirely new details [27]. Moreover, blink frequency, total number of blinks, maximum blink duration, and pupil diameter are positively correlated with mental workload [28]. Thus, eye tracking provides insight into information processing during intensive tasks as the user manipulates interface features. Functional evaluation of the interface is combined with finding the best functionality to achieve the best design [29]. Eye-tracking metrics such as first fixation time, total fixation time, and total number of fixations allow detailed measurement of the psychological characteristics of online cognitive processes [30-32].

### Study Aims

The aim of this study was to investigate how sleep diary interface design in a BBT-I app influences users’ attention, visual attention patterns, and short-term memory. Three sleep diary interfaces with systematically varied visual layouts, color schemes, box shapes, and input modalities were developed and evaluated. A mixed methods approach was used: objective eye-tracking measures were used to assess distribution of attention and task efficiency during interface interaction, whereas subjective attention scales, memory recall tasks, and user preference questionnaires captured cognitive performance and user experience. The study further examined how insomnia severity, SlpQ, and related symptoms moderated these outcomes. It was expected that interfaces with lower cognitive load and clearer interaction structures would support more stable attention, higher memory accuracy, and greater user satisfaction, particularly among individuals with stronger insomnia-related cognitive vulnerability.

## Methods

### Introduction to Sleep App Functions

The functions of our app were developed according to the requirements of BBT-I. Under the guidance of specialist clinical psychologists and physicians, patients with insomnia were asked to complete a sleep diary and the Dysfunctional Beliefs and Attitudes about Sleep: Validation of a Brief Version-16, which helped therapists to diagnose and understand the distinct causes and types of insomnia for each patient. According to the type of insomnia, a sleep-aid behavioral plan was tailored, including sleep hygiene education, stimulus control, and sleep restriction. The content of sleep hygiene education included paying attention to the sleeping environment, adjusting indoor temperature and humidity, timing of caffeine intake, ensuring sufficient physical activity during the day, and controlling the length of naps.

**Figure 1.**
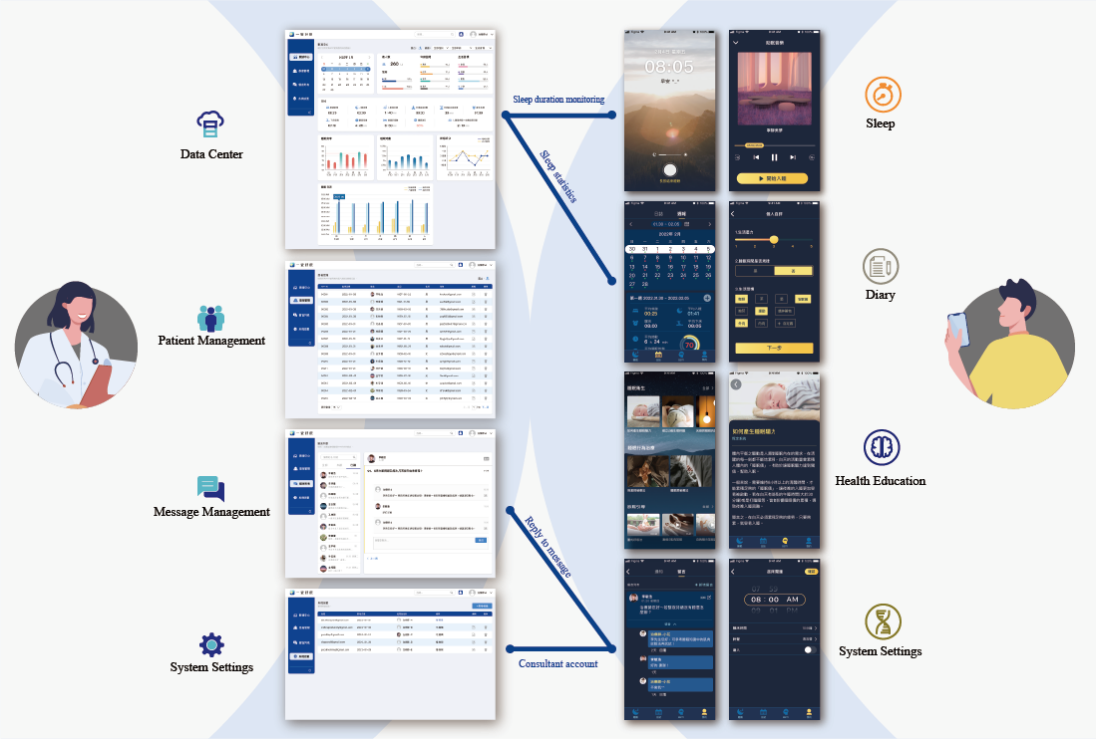
Association diagram of the front-end and back-end components of “Sleep Well.”

In this study, we developed an app front-end and back-end management system named “Sleep Well.” The front-end structure for patients consisted of the following components: (1) sleep timer, (2) sleep diary, (3) BBT-I health education, and (4) system settings. The sleep timer calculated bedtime and wake-up time. If the patient was unable to fall asleep, they could choose relaxation guidance videos or sleep-inducing music for assistance. The sleep diary was used to record various sleep-stage times, daytime habits from the previous day, and self-assessments of SlpQ and mental state (MS) after waking up. BBT-I health education conveyed correct knowledge related to sleep, which consisted of sleep hygiene, sleep behavior therapy, relaxation guidance, and sleep-inducing music. System settings included basic settings and message functions. During treatment, users could consult and interact online with dedicated therapists through the messaging area, and therapists could observe patients’ usage status in the background (Figure 1). The back-end structure of the app consisted of the following components: (1) data center, (2) patient management, (3) message management, and (4) system settings. The data center collected statistics on physiological characteristics, lifestyle habits, recovery time, and sleep conditions of all patients with insomnia, enabling an understanding of the treatment results for a certain period or a certain group of people. Patient management reviewed daily sleep data, diary records, and consultation results. Message management was used by patients to send messages and by therapists to respond to patient queries. System settings contained therapist account management, permissions, and notification settings.

### Sleep Diary: Three Interface Design Features

Sleep diaries primarily record the sleep time and daily routine of patients with insomnia, providing therapists with an assessment of the factors affecting sleep and planning sleep hours for patients.

**Figure 2.**
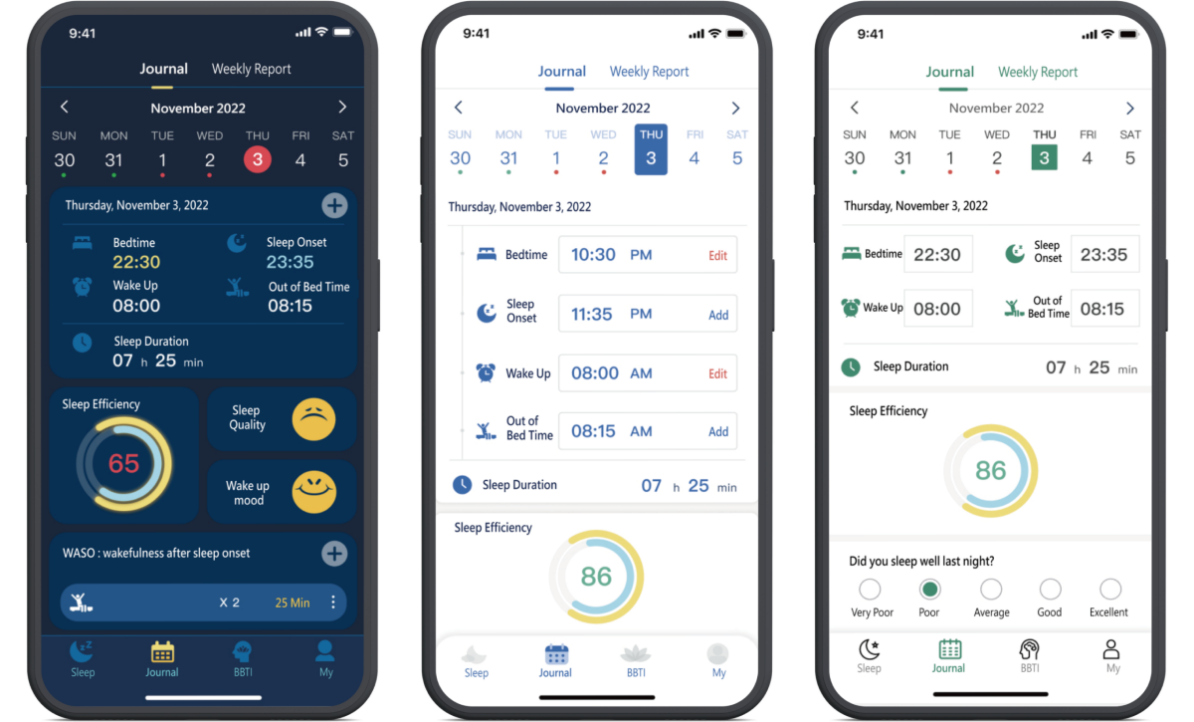
Visual comparison of sleep diary interfaces (from left to right: interfaces A, B, and C)*.*

This study extracted the core function of the app (ie, the sleep diary record page) and extended it into 3 different interface designs by varying background (day vs night), color scheme, box shape, input information, and interactive operation methods. Interface A used night mode with a blue color scheme, circular boxes, and a card-style appearance; the time input format was mainly sliding. Interface B used day mode with a blue color scheme, rounded rectangular box, a pop-up appearance, and tap-based input operation. Interface C used day mode with a green color scheme, rectangular boxes, a keyboard-based appearance, and direct manual input (tape-in) interaction (Figure 2).

The day-night background used a light background to imply daytime and a dark background to simulate nighttime. Matching icon colors and box shapes could stimulate the users’ feelings toward the interface, and the input information and interactive operation methods could measure operation preferences. Furthermore, participants completed subjective questionnaires, and an eye tracker was used to measure the gaze duration, gaze position, saccade duration, number of saccades (NS), and saccade direction (SDir) to provide an integrated analysis of the factors that affected the differences in attention and memory for the 3 interfaces.

### Experiment Methods

In the first phase, participants completed standardized recall tasks using all 3 sleep diary interfaces (A, B, and C). The order of interface presentation was counterbalanced across participants using a Latin square design to control for potential order and learning effects, which are common concerns in within-subject experimental designs [33,34]. Each interface was operated twice, and both task accuracy and differences in completion time between the first and second trials were recorded to assess learning-related performance changes.

Eye-tracking data were collected during a predefined task round for each participant, with the specific interface assigned according to the counterbalanced sequence. This approach ensured consistent measurement conditions while minimizing fatigue and carryover effects across interfaces, in line with established eye-tracking methodology in human–computer interaction and cognitive task research [29,30,32].

### Primary and Secondary Outcomes

The primary outcome of this pilot study was attentional performance during sleep diary interface interaction. Attentional performance was assessed using both objective eye-tracking indicators, including task completion time, NS, gaze stability, and SDir, and subjective attentional control, measured by the Attention Control Scale (ATTC). These measures were selected to capture complementary aspects of visual attention allocation, cognitive load, and attentional regulation during interface use.

Secondary outcomes included short-term memory performance during diary recall tasks and interface preference and user experience. Memory outcomes were examined to explore downstream cognitive effects associated with attentional demands, whereas preference-related measures were included to inform practical interface design implications for digital BBT-I applications.

### Participant Recruitment and Eligibility Criteria

The study was conducted between April 1 and May 15, 2023. A total of 33 participants aged 20 to 63 years were voluntarily recruited through Taipei Medical University. All participants provided written informed consent and completed a self-administered questionnaire that included demographic information and the Insomnia Severity Index (ISI) [35]. Clinical insomnia was defined as meeting at least one of the following criteria more than 3 times per week for a minimum duration of 3 months: (1) difficulty initiating sleep, defined as taking more than 30 minutes to fall asleep after going to bed; (2) difficulty maintaining sleep, defined as being awake for more than 30 minutes after sleep onset (SlpOn); or (3) early morning awakening, defined as waking at least 30 minutes earlier than desired. In addition, participants with an ISI total score of 15 or higher were classified as having clinical insomnia [35]. Participants without clinically significant insomnia were defined as those with ISI total score of 14 or lower and no subjective complaints of insomnia symptoms.

### Data Collection

**Figure 3.**
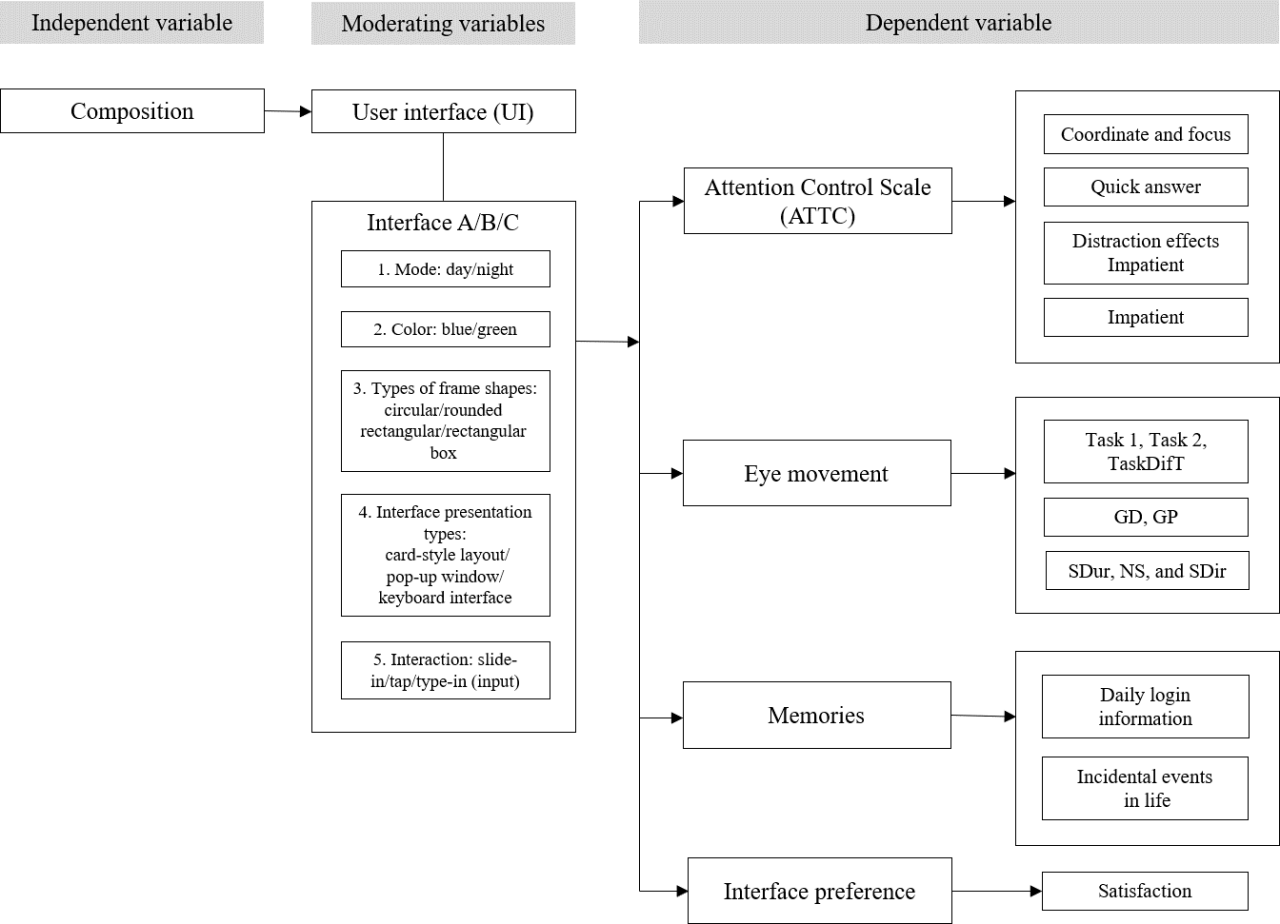
Correlations between population characteristics (independent variables) and 4 dependent variables (ATTC, eye movements, memory recall, and interface preference). GD: gaze duration; GP: gaze position; NS: number of saccades; SDir: saccade direction; SDur: saccade duration; TaskDifT: difference in task operation time.

Independent variables included demographic characteristics (personal characteristics, lifestyle habits, sleep assessment [ISI-1a to 1c items], and insomnia [ISI-2 to 5 items]) and a MS questionnaire were analyzed through interface characteristics (screen polarity, box design, and layout, and information input method) and 4 dependent variables: (1) interface preference, (2) subjective attention (including coordinate and focus, quick answer, distraction effects, and impatient), (3) eye movement data (operation time 1 [task 1], operation time 2 [task 2], difference in operation time [TaskDifT], gaze duration, gaze position, saccade duration, NS, and SDir), and (4) memories (daily login information and incidental events in life) (Figure 3). All study variables were categorized into conceptual domains, including population characteristics, lifestyle factors, sleep assessments, eye-tracking indicators, and memory recall measures. A complete classification of variables by name and type is provided in Multimedia Appendix 1.

We examined whether the demographic characteristics in the 4 dimensions contained other factors that could influence a user’s operation. Subjective attention measured the mentality of a user and their response to the operating interface. The eye tracker presented the attentiveness of a user and other physiological signals. Recall involved short-term and long-term memory as well as cognitive load.

Age, gender, lifestyle factors, sleep status (sleeping <7 h or below average sleep duration), severity of insomnia, and attention level were collected based on questionnaires and attention scales (Table 1). Eye movements were measured using an eye tracker (GP3 webcam, Gazepoint), and subjective opinions of the users for all 3 interfaces were collected through a postquestionnaire.

The recorded variables of the app operation were recall tasks that required inputting sleep information daily, including SlpOn, out-of-bed time, and wake after sleep onset (WASO; life events such as drinking (drinking items and drinking time [DrkTi]); exercise items (ExcsIm), exercise start time, and exercise end time, and nap start time and nap end time.

**Table 1.** Factor classification of the Attention Control Scale, with ATN^a^ denoting item numbers, based on exploratory factor analysis.

Classification and question	Content	Detailed description
Coordinate and focus		
ATN5	Difficulty in coordinating recall	When filling out the sleep diary, I find it difficult to coordinate attention between completing optional information and recalling the task description
ATN6	Ease of responding after distraction	After being interrupted or distracted, I can easily refocus on the content I am filling out
Quick answer		
ATN2	Difficulty recalling information quickly	It is difficult to quickly recall the description of the task
ATN3	Filling in the form to generate interest	When I have a sleep task, I quickly become interested in filling out the sleep diary
ATN4	Time-consuming adaptation to operation	It takes me a while to get used to the mode of operation of filling out the sleep diary
Distraction effects		
ATN7	Unable to think simultaneously	It is impossible to think about two fields in the sleep diary at the same time
ATN8	Distraction from others	I easily become distracted when someone is in the same space as I am when filling out the sleep diary or doing a recollection
ATN9	Rapid switch mode	I can quickly switch from the previous interface operation mode to the current interface operation mode
ATN1	Inattention to surroundings	I can easily concentrate when filling out the sleep diary without being aware of what is happening around me
Impatient		
ATN10	Impatience while answering questions	I rush to answer the questions in the sleep diary before I finish reading them.

a ATN: Attention Control Scale item number.

### Ethical Considerations

The TMU Joint Institutional Review Board from Taipei Medical University approved this study, which was planned, carried out, analyzed, and interpreted independently of any industry partners (approval number N202302007). All participants provided voluntary, informed, and written consent. The capability of the patients to participate in the study was ensured. During data analysis, the information of the participants was pseudonymized and coded using identification numbers. Only researchers assigned to the study had access to the data. No compensation was provided to participants for their participation and no images of individual participants or identifiable features are included in the paper or Multimedia Appendix 2.

### Statistical Analysis

Statistical analyses were performed using IBM SPSS Statistics (version 26; IBM Corp). Analyses included descriptive statistics, nonparametric tests, Pearson product–moment correlation coefficient analysis, cross-tabulation analysis, and exploratory factor analysis. Each participant completed the questionnaire 3 times, corresponding to the 3 different interfaces. To control for multiple comparisons, the Bonferroni correction was applied. The significance level (*α*=.05) was divided by 3, resulting in an adjusted *P* value threshold of .017 for each individual test. Eye-tracking data were collected using the GP3 webcam eye tracker (Gazepoint). Recorded data were processed and analyzed using Gazepoint Analysis software, which is supported exclusively on the Windows operating system.

## Results

### Participant Characteristics

A total of 33 participants (n=13, 39.4% male and n=20, 60.6% female) of age 20 to 64 years completed this study. Based on the ISI, 27 out of 33 (81.8%) participants were classified as those without clinically significant insomnia, and 6 out of 33 (18.2%) were classified as having clinical insomnia. Based on subjective reports, 13 out of 33 (39.4%) participants were categorized as having self-reported insomnia (SRI), and 20 out of 33 (60.6%) were categorized as having no self-reported insomnia (NSRI). Demographic and sleep-related characteristics are summarized in Tables2  3.

In total, 22 out of 33 (66.7%) participants reported staying up late, including 12 NSRI and 10 SRI individuals. Daily tea drinking was reported by 17 out of 33 (51.5%) participants, of whom 11 were NSRI. Daily coffee drinking was reported by 15 out of 33 (45.5%) participants. In addition, 5 out of 33 (15.2%) participants were current smokers, 2 out of 33 (6.1%) reported alcohol drinking, 7 out of 33 (21.2%) reported long-term medication use, and 18 out of 33 (54.5%) reported having a regular sleep schedule.

**Table 2.** Cross-analysis of gender and age group (N=33).

Age group (y)	Male, n	Female, n	Total, n (%)
20‐29	4	6	10 (30.3)
30‐44	6	10	16 (48.5)
45‐64	3	4	7 (21.2)
Total, n (%)	13 (39.4)	20 (60.6)	33 (100)

**Table 3.** ISI^a^ and cross-analysis table of patients with SRI^b^ and NSRI^c^.

ISI score	NSRI, n (%)	SRI, n (%)	Total, n (%)
0-7 (no insomnia)	14 (70)	0 (0)	14 (42.4)
8-14 (mild insomnia)	6 (30)	7 (53.8)	13 (39.4)
15-21 (moderate insomnia)	0 (0)	5 (38.5)	5 (15.2)
22-28 (severe insomnia)	0 (0)	1 (7.7)	1 (3)
Total	20 (100)	13 (100)	33 (100)

a ISI: Insomnia Severity Index.

b SRI: self-reported insomnia.

c NSRI: no self-reported insomnia.

### Attentional Performance (Eye-Tracking Measures)

Task efficiency differed by demographic and sleep factors. Age was associated with longer task completion times on Interface B (*H*=8.943, *P*=.01) and Interface C (task 1: *H*=10.075, *P*=.006; task 2: *H*=12.472, *P*=.002). Gender differences were observed in saccade counts on Interface C (*H*=7.294, *P*=.007).

Sleep characteristics were consistently associated with eye tracking outcomes. Poorer SlpQ was associated with a higher NS on Interface B (*r*=−0.448, *P*=.01) and Interface C (*r*=−0.491, *P*=.005). Sleep maintenance difficulty (ISI-1b) was associated with longer optimal operation time on Interface A (*r*=0.462, *P*=.007) and increased NS on Interface C (*r*=0.430, *P*=.02). Worry about sleep (ISI-5) was negatively associated with operation time 2 (task 2) on Interface C (*r*=−0.426, *P*=.01), indicating altered task dynamics.

**Figure 4.**
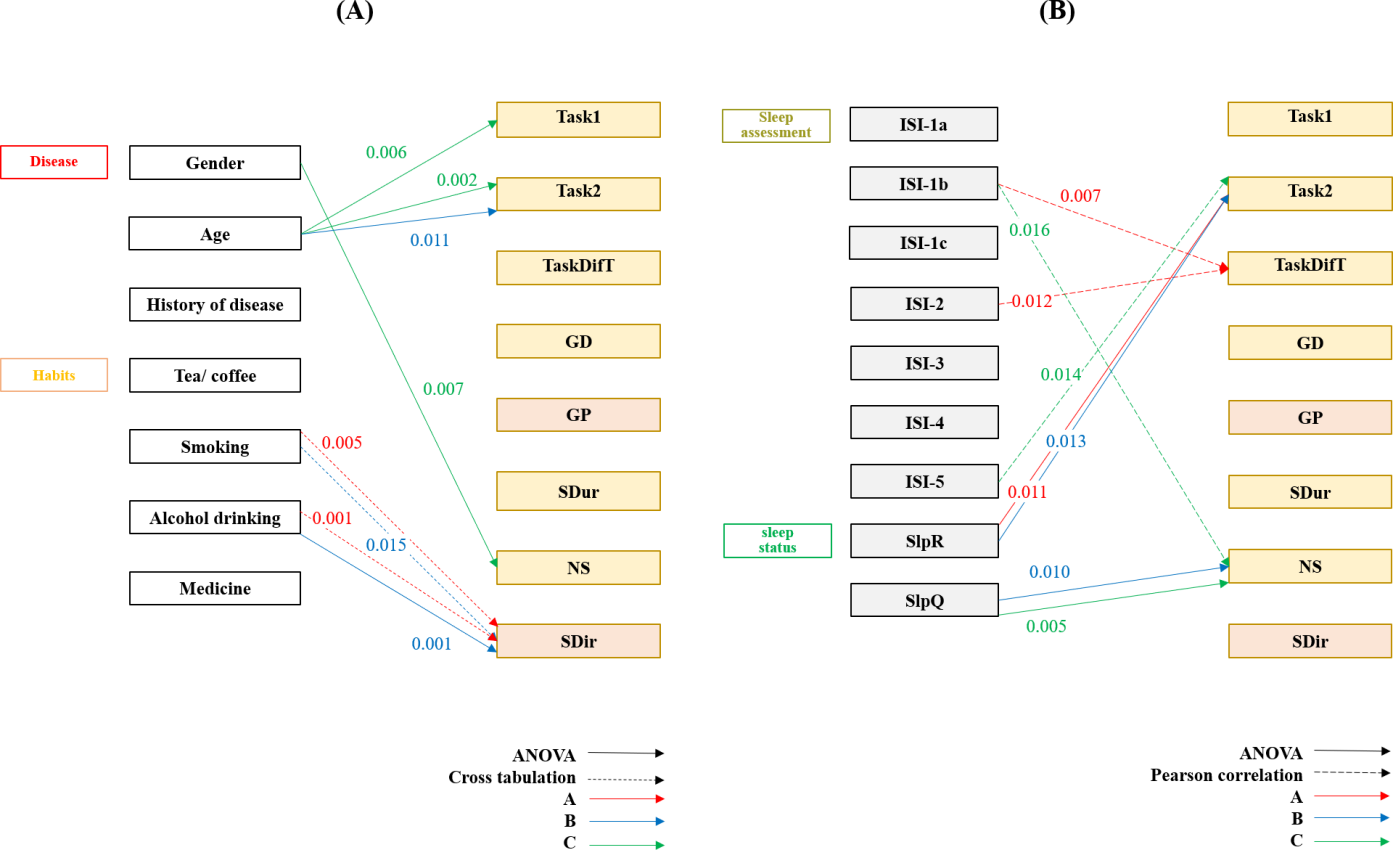
Relationships between (A) population characteristics and (B) insomnia symptoms (ISI) and eye-tracking information. GD: gaze duration; GP: gaze position; ISI: Insomnia Severity Index; NS: number of saccades; SDir: saccade direction; SDur: saccade duration; SlpQ: sleep quality; SlpR: sleep regularity; TaskDifT: difference in task operation time.

Lifestyle factors also influenced gaze behavior. Smoking and alcohol drinking were associated with SDir variability on Interfaces A and B (*χ*²=18.942‐28.562, *P*≤.02; Figure 4).

Eye-tracking results indicated that visual attention allocation and task efficiency differed across interfaces. Older age was consistently associated with longer task operation times, particularly on Interfaces B and C, indicating age-related reductions in interaction efficiency. Gender differences were observed in NS in Interface C, suggesting distinct visual scanning strategies.

Sleep-related factors played a central role in attentional performance. Poorer SlpQ and irregular sleep patterns were associated with increased NS and longer operation times, reflecting higher cognitive workload during interface interaction. Lifestyle behaviors, including smoking and alcohol drinking, were associated with altered SDir on Interfaces A and B, indicating changes in visual exploration patterns.

Among the 3 designs, Interface A (night mode, circular boxes, sliding input) was characterized by fewer saccades and more stable gaze transitions, suggesting reduced attentional dispersion and lower cognitive demand during task execution.

### Short-Term Memory Performance

Age was significantly associated with the task of remembering ExcsIm in Interface A (*H*_2_=11.886, *P*=.003).

Alcohol drinking showed significant effects on the task of DrkTi in Interface B (*H*_2_=10.551, *P*=.005) and the task of ExcsIm in Interface B (*H*_2_=15.500, *P*<.001). Staying up late was also associated with drinking items in Interface B (*H*_1_=6.400, *P*=.01).

Current sleep status was related to memory accuracy (total) on Interface A (*r*=–0.505, *P*=.003) and Interface A (*r*=–0.558, *P*<.001), as well as nap end time on Interface B (*r*=–0.468, *P*=.006).

**Figure 5.**
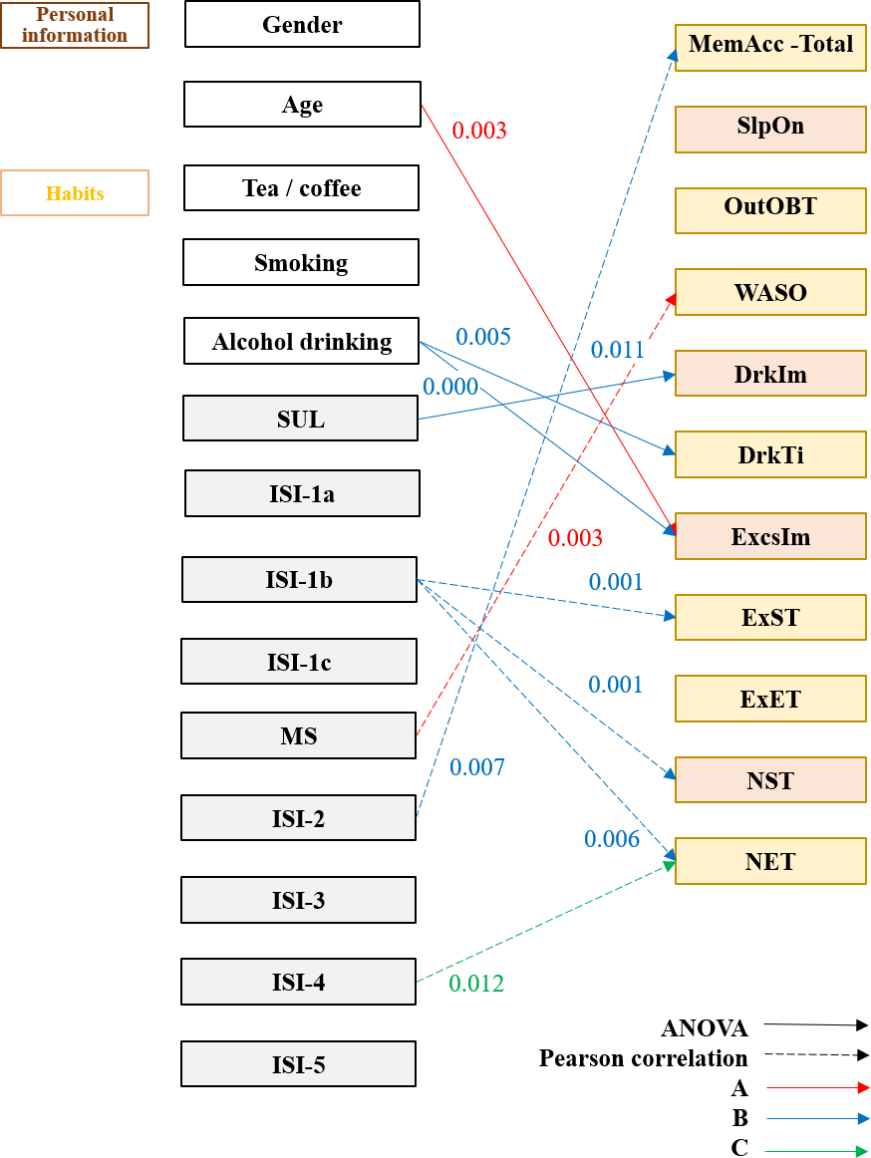
Relationships between population characteristics, insomnia symptoms (ISI), and memory recall scores. DrkIm: drinking items; DrkTi: drinking time; ExcsIm: exercise items; ExET: exercise end time; ExST: exercise start time; ISI: Insomnia Severity Index; MemAcc-Total: total memory accuracy; MS: mental state; NET: nap end time; NST: nap start time; OutOBT: out-of-bed time; SlpOn: sleep onset; SUL: stay up late; WASO: wakefulness after sleep onset.

ISI-2 was associated with memory accuracy total (*r*=–0.407, *P*=.007), and ISI-4 was associated with DrkTi in Interface C (*r*=–0.431, *P*=.01; Figure 5)

### Subjective Attentional Control (ATTC)

The Kruskal-Wallis *H* test showed that age was significantly associated with ATN6 (ATTC item number 6) on Interface B (*H*=10.643, *P*=.005). Among lifestyle factors, smoking was significantly associated with ATN1 on Interface B (*H*=8.585, *P*=.01). In addition, tea drinking was significantly associated with ATN6 on both Interface B (*H*=10.641, *P*=.01) and Interface C (*H*=10.519, *P*=.02).

**Figure 6.**
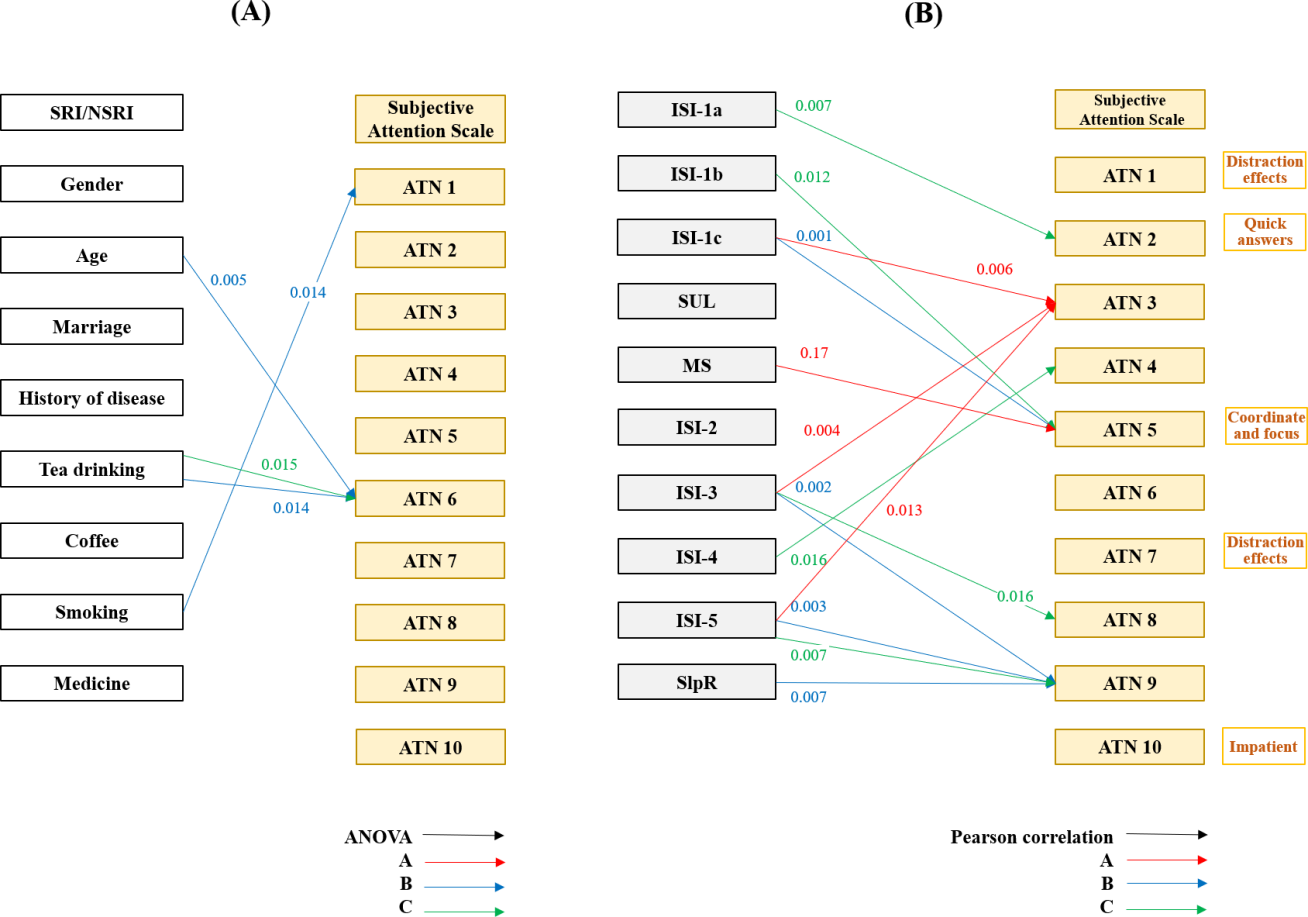
Associations between (A) population characteristics and (B) insomnia symptoms (ISI) and subjective attention scale across interfaces. ATN: Attention Control Scale item number; ISI: Insomnia Severity Index; MS: mental state; NSRI: no self-reported insomnia; SlpR: sleep regularity; SRI: self-reported insomnia; SUL: stay up late.

Associations were observed between sleep-related factors, insomnia symptoms, and specific subjective attention (ATN) domains across interfaces. Sleep regularity was positively associated with ATN9 (distraction effects) on Interface B (*r*=0.460, *P*=.007). Regarding sleep initiation difficulties, ISI-1a was positively associated with ATN2 (quick answers) on Interface C (*r*=0.461, *P*=.007), and ISI-1b was positively associated with ATN5 (coordinate and focus) on Interface C (*r*=0.434, *P*=.01). Early morning awakening (ISI-1c) was positively associated with ATN3 (quick answers) on Interface A (*r*=0.467, *P*=.006) and with ATN5 (coordinate and focus) on Interface B (*r*=0.563, *P*<.001). In contrast, sleep interference with daily functioning (ISI-3) was negatively associated with ATN3 (quick answers) on Interface A (*r*=−0.487, *P*=.004), but positively associated with ATN9 (distraction effects) on Interface B (*r*=0.528, *P*=.002) and ATN8 (distraction effects) on Interface C (*r*=0.417, *P*=.02). ISI-4 showed a positive association with ATN4 (quick answer) on Interface C (*r*=0.417, *P*=.02). Worry about sleep (ISI-5) was negatively associated with ATN3 (quick answers) on Interface A (*r*=−0.426, *P*=.01), but positively associated with ATN9 (distraction effects) on Interface B (*r*=0.505, *P*=.003) and Interface C (*r*=0.463, *P*=.007). In addition, MS was negatively associated with ATN5 (coordinate and focus) on Interface A (*r*=−0.412, *P*=.02; Figure 6).

Specific ATTC subdomains were selectively associated with demographic and lifestyle factors. Age was related to attention refocusing ability in Interface B, while smoking and tea drinking were associated with distraction-related indices.

Insomnia symptoms demonstrated robust associations with subjective attention. Greater sleep initiation and maintenance difficulties, early morning awakening, and daytime dysfunction were associated with increased distractibility and reduced attentional coordination across interfaces. Worry about sleep was consistently associated with impulsive responding and reduced sustained attention.

MS showed a negative association with attentional coordination, highlighting the interaction between emotional condition and cognitive control during interface use.

### Interface Preference and User Experience

Under normal daylight conditions, no significant differences were observed between SRI and NSRI participants in preference for night versus day mode. NSRI participants showed a greater preference for circular boxes (*χ*²_1_=6.421, *P*=.01), whereas SRI participants preferred rounded rectangular boxes (*r*=0.424, *P*=.01). Insomnia symptoms participants reported higher satisfaction with Interface A (*χ*²_12_=27.094, *P*=.007), while those with clinical insomnia showed a lower preference for rounded rectangular boxes (*r*=0.593, *P*<.001).

The ISI total score was positively correlated with preference for rounded rectangular box (*r*=0.431, *P*=.01). ISI-1b was associated with rectangular box (*r*=0.440, *P*=.01), whereas ISI-3 and 4 were associated with rounded rectangular boxes (*r*=0.498, *P*=.003; *r*=0.437, *P*=.01). ISI-5 was associated with indifference toward interface interaction design (*r*=0.439, *P*=.01). Wake-up time was negatively correlated with preference for type-in (*r*=−0.479, *P*=.005), and sleep duration was positively correlated with preference for night mode (*r*=0.414, *P*=.02).

**Figure 7.**
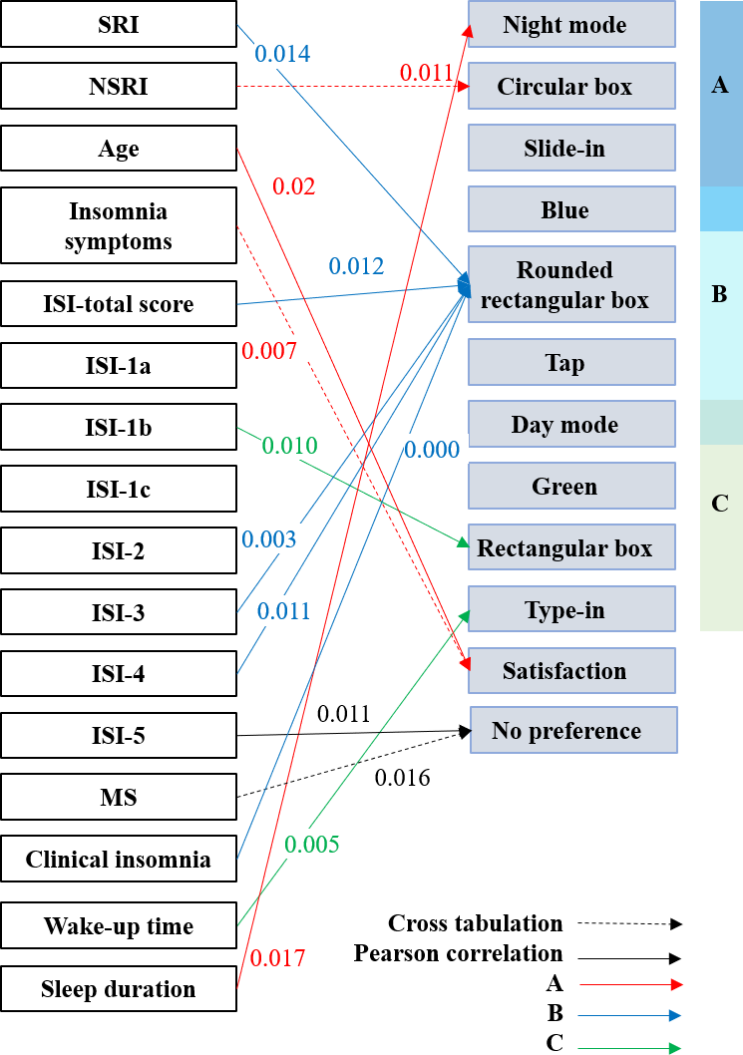
Pearson correlation and cross-tabulation analyses of insomnia measures and interface preferences. ISI: Insomnia Severity Index; MS: mental state; NSRI: no self-reported insomnia; SRI: self-reported insomnia.

Between MS and the interface interaction mode, there was no preference (*χ*²_3_=10.313, *P*=.02; Figure 7).

Using K-means clustering, participants were first divided into 2 clusters based on those without clinically significant insomnia and those with clinical insomnia to examine differences in preferences among Interfaces A, B, and C. Significant between-cluster differences were observed in satisfaction with Interface A (*F*_1,31_=18.164, *P*<.001), overall interface satisfaction (*F*_1,31_=7.691, *P*=.009), interaction duration (*F*_1,31_=38.214, *P*<.001), and consistency of interface interaction (*F*_1,31_=44.574, *P*<.001). Day mode also differed significantly between clusters (*F*_1,31_=10.333, *P*=.003).

A second K-means analysis divided participants into 4 clusters based on insomnia symptoms. Significant cluster effects were found for satisfaction with Interface A (*F*_3,29_=4.726, *P*=.008), Interface B (*F*_3,29_=11.824, *P*<.001), and Interface C (*F*_3,29_=6.519, *P*=.002). Clusters also differed significantly in interaction duration (*F*_3,29_=5.800, *P*=.003), overall interface satisfaction (*F*_3,29_=15.501, *P*<.001), and consistency of interface interaction (*F*_3,29_=36.099, *P*<.001). In addition, significant differences were observed in preferences for color modes (eg, color mode 1: *F*_3,29_=3.643, *P*=.02; color mode 2: *F*_3,29_=4.233, *P*=.01) and interaction methods, including slide-in (*F*_3,29_=3.522, *P*=.03) and tap input (*F*_3,29_=5.446, *P*=.004).

Participants without SRI showed a significantly greater preference for rounded rectangular visual designs and reported higher satisfaction with Interface A. In contrast, participants with clinical insomnia preferred rounded rectangular designs.

Sleep characteristics further influenced preferences. Longer sleep duration was associated with preference for night-mode displays. Cluster analyses based on insomnia status and symptom severity revealed significant differences in interface satisfaction, perceived task duration, and preferred interaction methods, with Interface A most frequently rated as the most efficient overall.

### Integrated Relationships Between Attention, Eye Tracking, and Memory

Across the 3 interfaces, several subjective attention (ATN) indicators showed significant negative associations with task memory performance during operational tasks. On Interface A, task memory performance was negatively correlated with ATN7 (distraction effects; *r*=−0.446, *P*=.009), ATN1 (distraction effects; *r*=−0.498, *P*=.003), and ATN10 (impatient; *r*=−0.456, *P*=.008), indicating that greater distraction and impatience were associated with poorer task recall. On Interface B, a significant negative association was observed between task memory performance and ATN5 (coordinate and focus; *r*=−0.410, *P*=.02), ATN2 (quick answers; *r*=−0.564, *P*<.001), and ATN7 (distraction effects; *r*=−0.477, *P*=.005), suggesting that reduced attentional coordination, quick answers, and distraction were related to lower recall accuracy. On Interface C, task memory performance was negatively correlated with ATN7 (distraction effects; *r*=−0.488, *P*=.004), ATN1 (distraction effects; *r*=−0.511, *P*=.002), and ATN5 (coordinate and focus; *r*=−0.430, *P*=.01; Table 4).

**Table 4.** Associations between subjective attention (ATN^a^) indicators and task memory performance across interfaces.

Interface	ATN indicator (domain)	*r*	*P* value
A	ATN7 (distraction effects)	−0.446	.009^b^
A	ATN1 (distraction effects)	−0.498	.003^b^
A	ATN10 (impatient)	−0.456	.008^b^
B	ATN5 (coordinate and focus)	−0.410	.02
B	ATN2 (quick answers)	−0.564	<.001^b^
B	ATN7 (distraction effects)	−0.477	.005^b^
C	ATN7 (distraction effects)	−0.488	.004^b^
C	ATN1 (distraction effects)	−0.511	.002^b^
C	ATN5 (coordinate and focus)	−0.430	.01

a ATN: Attention Control Scale item number.

b *P*<.02.

Overall, higher levels of subjective attentional difficulty (eg, distraction, impatience, reduced coordination) were consistently associated with lower task memory accuracy. Eye-tracking measures supported these findings: faster operation times and more stable saccade patterns were associated with better recall of time-based diary entries.

These convergent results indicate that interface designs that reduce attentional fragmentation also facilitate more accurate short-term memory during sleep diary completion.

Associations were observed between eye-tracking metrics, task memory performance, and subjective attention (ATN) indicators across interfaces. On Interface A, SDir was negatively correlated with SlpOn (*r*=−0.475, *P*=.006) and significantly associated with WASO (*H*_4_=14.500, *P*=.006), indicating that greater directional variability in gaze was related to longer memory-encoding time and nocturnal sleep fragmentation. On Interface B, task 2 was negatively correlated with memory recall duration (*r*=−0.411, *P*=.02), suggesting improved task efficiency during secondary operations. With respect to subjective attention, task 2 on Interface B was negatively correlated with ATN1 (distraction effects; *r*=−0.415, *P*=.02), whereas on Interface A, the NS was positively correlated with ATN8 (distraction effects*; r*=0.424, *P*=.02). Collectively, these findings indicate that interface-specific eye-movement patterns are associated with both cognitive workload during task execution and subjective attentional control, with gaze stability reflecting more efficient memory processing and reduced attentional disruption.

**Figure 8.**
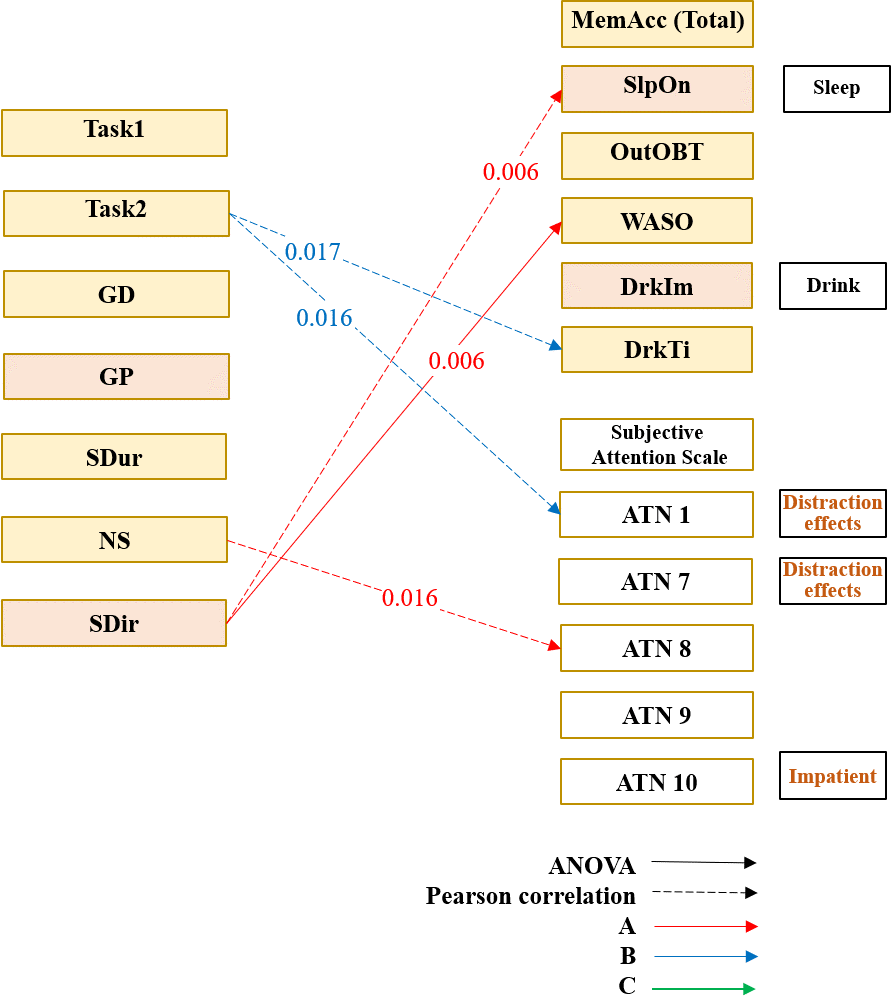
Associations between eye-tracking metrics, task memory performance, and subjective attention. ATN: Attention Control Scale item number; DrkIm: drinking items; DrkTi: drinking time; GD: gaze duration; GP: gaze position; MemAcc-Total: total memory accuracy; NS: number of saccades; OutOBT: out-of-bed time; SDir: saccade direction; SDur: saccade duration; SlpOn: sleep onset; WASO: wakefulness after sleep onset.

Eye-tracking metrics showed selective associations with memory: greater SDir variability on Interface A was associated with longer sleep-onset recall (*r*=−0.475, *P*=.006) and WASO (*H*_4_=14.500, *P*=.006). Faster second-trial task times on Interface B were associated with improved recall (*r*=−0.411, *P*=.02; Figure 8).

Detailed statistical results for all nonparametric and correlation analyses are provided in Multimedia Appendix 1.

## Discussion

### Principal Results

This study investigated how interface design features of a BBT-I sleep diary app influence attention, visual processing, and short-term memory in individuals with varying insomnia severity. By integrating eye-tracking metrics, subjective attention ratings, behavioral memory accuracy, and interface preferences, the study provides a multidimensional understanding of how insomnia-related cognitive vulnerabilities interact with digital interface design.

Interface design was associated with differences in objective attentional performance, as reflected in task completion time, saccade count, and gaze stability. Second, SlpQ and insomnia symptoms—particularly sleep maintenance difficulty and sleep-related worry—were consistently associated with impaired attention and reduced memory accuracy. Third, short-term memory performance varied across interface designs and was especially sensitive to sleep disruption during event-based recall. Fourth, subjective attentional control showed strong convergence with both eye-tracking indicators and memory outcomes. Finally, interface preferences differed by insomnia status, with participants without clinically significant insomnia favoring visually softer, lower-load designs.

### Interface Design and Attentional Load

Differences in eye-movement patterns across interfaces indicate that layout structure and interaction modality directly shape attentional demands. Interfaces requiring more visual scanning or manual input elicited higher saccade activity and longer task durations, particularly among participants with poorer SlpQ. In contrast, designs characterized by simpler layouts and guided interactions were associated with more stable gaze patterns and reduced attentional dispersion. These findings support cognitive load theory, suggesting that insomnia amplifies sensitivity to interface complexity and visual fragmentation.

### Insomnia Symptoms and Memory Vulnerability

Memory accuracy was particularly vulnerable to sleep maintenance difficulties and daytime dysfunction, especially for event-based diary items such as exercise and nap timing. Routine sleep information (eg, bedtime and wake time) was less affected. This selective impairment aligns with prior evidence that insomnia disproportionately affects episodic and temporally ordered recall. Interface designs that increase cognitive effort during data entry may therefore exacerbate memory errors in users with insomnia.

### Subjective Attention as a Key Predictor

One of the most robust findings was that subjective attentional difficulties—such as distraction, impatience, and reduced coordination—were stronger predictors of memory accuracy than eye-tracking measures. While eye-tracking captured physiological effort and scanning behavior, subjective attention better reflected the internal cognitive state governing successful recall. This suggests that memory recall during sleep diary completion is primarily an internally driven cognitive process, rather than one dependent on overt visual behavior.

### Limitations

The following limitations should be considered when interpreting the results of this study:

1. Our findings represented regional population characteristics. The participants were of Asian descent between the ages of 20 and 64. Their lifestyle habits and insomnia conditions may have been influenced by factors such as age, occupation, and region of residence.
2. One limitation of the present study was the difficulty in recruiting participants with insomnia, which led to an underrepresentation of individuals with severe insomnia. Future studies with larger samples are warranted to enhance statistical power and generalizability.
3. The sleep diary interface of the app used in our study was based on the log recording method of CBT-I, and we only investigated user experience as well as the preference, attention, and memory status of participants. The therapeutic effect of our interfaces on insomnia was not within the scope of our study.
4. The mobile device used was running iOS. Other mobile application systems would require additional designs.

### Conclusions

This study demonstrates that interface design plays a critical role in shaping attention, visual processing, and short-term memory performance in users with insomnia. Insomnia symptoms—particularly sleep maintenance difficulty and sleep-related worry—were consistently associated with attentional disruption and reduced memory accuracy during sleep diary completion. Notably, subjective attentional control was a stronger and more reliable predictor of memory performance than eye-tracking measures, indicating that cognitive processing during recall is driven primarily by internal attentional states rather than observable gaze behavior. For designers of digital insomnia interventions, these findings highlight the need to reduce cognitive load through simple, structured layouts; prioritize low-effort input methods such as tapping over typing; support sequential recall with guided, step-by-step data entry; and use visually soft, predictable designs that minimize distraction. Interfaces should also be adaptable to insomnia severity, offering more guided and low-complexity interactions for users with greater sleep disruption. By aligning interface design with the cognitive characteristics of insomnia, digital BBT-I applications may improve usability, data accuracy, and user engagement.

## Supplementary material

10.2196/79883 Multimedia Appendix 1 Classification of variables by name and type.

10.2196/79883 Multimedia Appendix 2 All statistical results.

10.2196/79883 Checklist 1 CONSORT 2010 checklist.

## Abbreviations

ATN: Attention Control Scale item number
ATTC: Attention Control Scale
BBT-I: brief cognitive behavioral therapy for insomnia
CBT-I: cognitive behavioral therapy for insomnia
CONSORT: Consolidated Standards of Reporting Trials
DrkTi: drinking time
ExcsIm: exercise items
ISI: Insomnia Severity Index
MS: mental state
NS: number of saccades
NSRI: no self-reported insomnia
SDir: saccade direction
SlpOn: sleep onset
SlpQ: sleep quality
SRI: self-reported insomnia
WASO: wake after sleep onset
