# Supplementary material for: Designing App Interfaces to Elicit Specific Emotional Responses and Improve Attention and Short-Term Memory in Patients With Insomnia Undergoing Brief Cognitive Behavioral Therapy: Within-Subject Eye-Tracking Experimental Pilot Study
Source: JMIR Hum Factors. 2026 Feb 19;13:e79883. doi: 10.2196/79883 (PMC12919966; doi:10.2196/79883)
Supplement: Multimedia Appendix 1 [file humanfactors-v13-e79883-s001.pdf]

Classification of variables by name and type.

Item	Variable Item	Variable
**population characteristics**	descriptive phrase / personal information	Insomnia, Gender, Age, Marriage, History of disease
	lifestyle	Tea, coffee, smoking, drinking, medicine
**user interface**	Night mode, Day mode, Circular box, Slide-in, Blue, Rounded Rectangular box, Tap, Green, Rectangular box, Type-in, Preference	
**sleep assessment**	ISI-1a	
	ISI-1b	
	ISI-1c	
	SUL	
**Current state**	MS	
**Interference**	ISI-2	
	ISI-3	
	ISI-4	
	ISI-5	
**sleep Status**	SlpR	
	SlpQ	
**Eye tracking information**	Task1	
	Task2	
	TaskDifT	
	GD	
	GP	
	SDur	
	NS	
	SDir	
**Memory recall**	MemAcc-Total	
	SlpOn	
	OutOBT	
	WASO	
**Drink**	DrkIm	
	DrkTi	
**Exercise**	ExcsIm	
	ExST	
	ExET	
**Nep**	NST	
	NET	
