# Supplementary material for: Designing App Interfaces to Elicit Specific Emotional Responses and Improve Attention and Short-Term Memory in Patients With Insomnia Undergoing Brief Cognitive Behavioral Therapy: Within-Subject Eye-Tracking Experimental Pilot Study
Source: JMIR Hum Factors. 2026 Feb 19;13:e79883. doi: 10.2196/79883 (PMC12919966; doi:10.2196/79883)
Supplement: Multimedia Appendix 2 [file humanfactors-v13-e79883-s002.pdf]

1. Kruskal–Wallis H test results showing factors that significantly influenced attention indicators.

Item	factors	A/B/C interface	Attention Control Scale (ATTC)	Statistical results
Population characteristics	age	B	ATN6	H = 10.643, p = .005
lifestyle	smoking	B	ATN1	H = 8.585, p = .014
	(tea drinking	B	ATN6	H = 10.641, p = .014
		C	ATN6	H = 10.519, p = .015

2. Correction Between Population Characteristics, Insomnia symptoms (ISI) and Subjective Attention scale Across Interfaces

Sleep-related factors	A/B/C interface	Attention Control Scale (ATTC)	Statistical results
Sleep regularity	B	ATN9	r = 0.460, p = .007
ISI-1a	C	ATN2	r = 0.461, p = .007
ISI-1b	C	ATN5	r = 0.434, p = .012
ISI-1c	A	ATN3	r = 0.467, p = .006
	B	ATN5	r = 0.563, p = .001
ISI-3	A	ATN3	r = -0.487, p = .004
	B	ATN9	r =0.528, p = .002
	C	ATN8	r =0.417, p = .016
ISI-4	C	ATN4	r = 0.417, p = .016
ISI-5	A	ATN3	r = -0.426, p = .013
	B	ATN9	r = 0.505, p = .003
	C	ATN9	r = 0.463, p = .007
Mental state (MS)	A	ATN5	r = -0.412, p = .017

3. Relationships between population characteristics, insomnia symptoms (ISI) and eye-tracking information.

Factor items	Influential variables	A/B/C interface	Affected eye-tracking indicators	Statistical results
Population characteristics	Gender	C	Number of Saccades	H = 7.294, p = .007
	Age	C	Operation time 1	H = 10.075, p = .006
		B	Operation time 2	H = 8.943, p = .011
		C	Operation time 2	H = 12.472, p = .002
lifestyle	Smoking	A	Saccade direction	χ^2 (10) = 25.135, p = .005
		B	Saccade direction	χ^2 (8) = 18.942, p = .015
	Alcohol drinking	A	Saccade direction	χ^2 (10) = 28.562, p = .001
		B	Saccade direction	χ^2 (8) = 26.070, p = .001
ISI Insomnia Severity	ISI-2b	A	Optimal time	r = 0.462, p = .007
		C	Number of Saccades	r=0.430, p=0.016
	ISI-2	A	Optimal time	r=0.433, p=0.012
	ISI-5	C	optime2	r=-0.426, p=0.014
sleep Status	Sleep quality	B	Number of Saccades	r = –0.448, p =0.010
		C	Number of Saccades	r=-0.491, p=0.005
	Sleep regularity	A	Operation time2	H (1) =6.411, p=.011
		B	Operation time2	H (1) =6.228, p=.013)

4. Relationships between population characteristics, insomnia symptoms (ISI) and memory recall scores.

Factor items	Variables	A/B/C interface	Task of memory	Statistical results
Population characteristics	Age	A	Exercise items (ExcsIm)	H (2) = 11.886, p = .003
lifestyle	Alcohol drinking	B	Drinking time (DrkTi)	H (2) = 10.551, p = .005
		B	Exercise items (ExcsIm)	H (2) =15.500, p=.000
	Stay up late (SUL)	B	Drinking items (DrkIm)	H (1) =6.400, p=.011
Current state	Mental state (MS)	A	wakefulness after sleep onset (WASO)	r=-0.505, p=0.003
ISI Insomnia Severity	ISI-2b	B	Exercise start time (ExST)	r=-0.550, p=.001
		B	Nap start time (NST)	r=-0.558, p=0.001
		B	Nap end time (NET)	r=-0.468, p=0.006
	ISI-2	B	MemAcc-Total	r=-0.463, p=0.007
	ISI-4	C	Nap end time (NET)	r=0.431, p=.012

5. Relationships Between Interface, Eye Tracking, and Memory

A/B/C interface	Affected eye-tracking indicators	Task of memory	Statistical results (r / p)
A	Saccade direction	Sleep Onset (SlpOn)	r = –.475, p = .006
A	Saccade direction	Out of Bed Time (OutOBT)	H (4) =14.500, p=.006
B	Operation time 2	Drinking time (DrkTi)	r = –.411, p = .017

6. **Relationships Between Interface, Eye Tracking, and Attention**

A/B/C interface	Affected eye-tracking indicators	Attention Control Scale (ATTC)	Statistical results (r / p)
B	Operation time 2	ATN1	r = –.415, p = .016
A	Saccade count	ATN8	r = .424, p = .016

7. Correlations among population characteristics, insomnia symptoms (ISI) and interface information.

Variable	Color-blue	Color-green	Box shape-circular	Box shape-round rectangular	Box shape-rectangular	No preference for shape	Slide-in	Tap	Type-in	No preference for input	Satisfaction-A	Background-night
Self-reported insomnia												
r	-0.065	0.268	-.441*	.424*	-0.039	0.143	-0.127	0.011	0.038	0.219	-0.037	-0.142
P-value (two-tailed test)	0.721	0.132	0.01	0.014	0.828	0.427	0.482	0.95	0.835	0.22	0.837	0.43
Age												
r	0.124	-0.019	-0.111	-0.244	0.177	0.213	-0.274	-0.2	0.27	-0.131	.403*	-0.184
P-value (two-tailed test)	0.493	0.915	0.54	0.171	0.323	0.234	0.123	0.264	0.129	0.468	0.02	0.304
insomnia symptoms												
r	0.1	-0.186	0.331	-.378*	0.145	-0.13	0.02	0.004	0.136	-0.275	0.094	0.01
P-value (two-tailed test)	0.581	0.3	0.06	0.03	0.422	0.472	0.914	0.982	0.451	0.121	0.601	0.956
ISI-total score												
r	-0.029	0.152	-0.326	.431*	0.289	-0.192	-0.23	-0.001	0.09	0.218	-0.063	-0.166
P-value (two-tailed test)	0.871	0.397	0.064	0.012	0.103	0.285	0.198	0.995	0.618	0.222	0.728	0.355
ISI-1a												
r	0.012	-0.013	-0.243	0.111	0.129	0.087	-0.118	0.121	0.023	0.134	-0.249	-0.035
P-value (two-tailed	0.949	0.941	0.172	0.537	0.473	0.629	0.512	0.503	0.899	0.458	0.162	0.846

test)												
ISI-1b												
r	-0.028	0.208	-0.093	-0.099	.440*	-0.092	0.049	-0.224	0.096	-0.194	-0.025	-0.158
P-value (two-tailed test)	0.878	0.245	0.606	0.583	0.01	0.612	0.786	0.209	0.595	0.278	0.89	0.378
ISI-1c												
r	0.106	0.098	-0.295	0.32	0.215	-0.086	-0.209	-0.232	0.228	0.01	-0.09	-0.186
P-value (two-tailed test)	0.558	0.587	0.096	0.069	0.23	0.635	0.243	0.193	0.202	0.956	0.619	0.3
ISI-2												
r	0.023	0.027	-0.257	.359*	0	-0.025	-0.025	0	0.092	-0.067	0	-0.094
P-value (two-tailed test)	0.897	0.882	0.149	0.04	1	0.89	0.89	1	0.61	0.71	1	0.601
ISI-3												
r	-0.108	0.104	-0.215	.498**	0.058	-0.223	-0.108	0.01	-0.074	0.187	-0.057	-0.013
P-value (two-tailed test)	0.548	0.563	0.229	0.003	0.747	0.213	0.55	0.958	0.684	0.298	0.754	0.942
ISI-4												
r	-0.007	0.133	-0.239	.437*	0.333	-0.318	-0.318	0.134	0.093	.409*	-0.03	-0.197
P-value (two-tailed test)	0.969	0.462	0.181	0.011	0.058	0.071	0.071	0.458	0.607	0.018	0.871	0.271
ISI-5												
r	-0.127	0.168	-0.239	.375*	0.203	-0.184	-0.305	0.137	-0.008	.439*	0.127	-0.111

P-value (two-tailed test)	0.483	0.351	0.18	0.032	0.258	0.306	0.084	0.446	0.963	0.011	0.481	0.54
Mental state (MS)												
r	-.388*	0.055	0.162	-0.055	-0.037	-0.105	0.149	-0.028	-0.016	.357*	0.314	0.277
P-value (two-tailed test)	0.025	0.761	0.368	0.761	0.839	0.561	0.409	0.875	0.927	0.041	0.075	0.118
Clinical insomnia												
r	0.072	0.1	-0.246	.593**	0.124	-0.311	-0.311	0.043	0.3	.375*	-0.142	-0.219
P-value (two-tailed test)	0.689	0.58	0.168	0	0.491	0.078	0.078	0.812	0.09	0.032	0.431	0.22
Wake-up time												
r	-0.102	-0.126	0.007	-0.069	0.032	0.03	0.335	-0.043	-.479**	0.236	-0.221	.380*
P-value (two-tailed test)	0.57	0.484	0.968	0.704	0.86	0.869	0.057	0.812	0.005	0.186	0.217	0.029
Sleep duration												
r	-0.169	-0.031	0.027	-0.057	-0.08	0.069	0.069	0.157	-.382*	0.237	0.021	.414*
P-value (two-tailed test)	0.347	0.865	0.883	0.753	0.657	0.702	0.702	0.384	0.028	0.184	0.909	0.017
